# Whole-genome sequence of *Paenibacillus amylolyticus* strain W018, isolated from *Triticum aestivum* L. seeds, obtained using nanopore sequencing

**DOI:** 10.1128/MRA.00687-23

**Published:** 2023-09-25

**Authors:** Vladimir K. Chebotar, Maria S. Gancheva, Elena P. Chizhevskaya, Oksana V. Keleinikova, Maria E. Baganova, Alexander N. Zaplatkin, Kharon A. Husainov

**Affiliations:** 1 Laboratory of Microbial Technology, All-Russian Research Institute for Agricultural Microbiology, St. Petersburg, Pushkin, Russia; 2 Department of Genetics and Biotechnology, Faculty of Biology, Saint Petersburg State University, St. Petersburg, Russia; 3 Laboratory of Generic and Selection of Microorganisms, Chechen Research Institute of Agriculture, Chechen Republic, Russia; University of Arizona, Tucson, Arizona, USA

**Keywords:** endophytes, wheat seeds, *Paenibacillus amylolyticus*, whole-genome sequence

## Abstract

In this study, we performed nanopore sequencing of the genome of *Paenibacillus amylolyticus* strain W018, isolated from the seeds of winter wheat, cv. Bezostaya 100. The genome size is 7.07 Mb, with a GC content of 45.8%, and contains 8,190 genes.

## ANNOUNCEMENT

Several species from the genus *Paenibacillus* are known to be involved in atmospheric nitrogen fixation, solubilization of phosphate and potassium, and the production of phytohormones and antibiotics [reviewed in reference (1)]. Here, we report the complete genome sequence of *Paenibacillus amylolyticus* strain W018, isolated from the seeds of winter wheat (*Triticum aestivum* L.), cv. Bezostaya 100, grown on typical medium-sized low-humus chernozem soil in the mountain and forest zone, Vedeno region, Chechen Republic, Russia. Seeds were disinfected with 70% ethanol for 30 s, rinsed with sterile water two times, then sterilized with bleach solution (15% sodium hypochlorite) for 30 min with shaking on a rotary shaker (PSU 20i, Biosan, Latvia) at 180 rpm, washed seven times with sterile water, and placed on GMF medium (g/L: beef enzymatic hydrolysate—15.0; NaCl—9.0; agar—15.0, LLC NICF, Saint-Petersburg, Russia) plates at 28°C for 48 h. If no bacteria or fungi growth was observed, seeds in 5 mL of sterile water were crushed with a mortar and pestle under sterile conditions. Aliquots of 40 mL of the resulting suspension were plated onto GMF medium. Plates were incubated at 28°C for 2 days. Bacterial isolation was performed as previously described (2). Strain W018 was grown in Luria-Bertani (LB) medium at 30°C overnight. DNA was extracted from a single colony with the cetyltrimethylammonium bromide-NaCl method (3).

The library was prepared with a ligation sequencing kit (catalog number SQK-LSK109) and barcoded using the EXP-NBD104 and EXP-NBD114 kits (Oxford Nanopore, UK) according to the manufacturer’s instructions. Sequencing was performed using a MinION device with a 9.4.1 flow cell (Oxford Nanopore, UK) in the Core Centrum “Genomic Technologies, Proteomics and Cell Biology” at the All-Russia Research Institute for Agricultural Microbiology. Base calling was performed with Guppy v3.3.0 (4) in the high-accuracy mode, and read data was quality assessed with FastQC v0.11.9 (5). Oxford Nanopore Technologies generated a total of 13,081 reads, accounting for 139,549,745 bases with a mean length of 10,668 bp and 16.5× coverage. The genome coverage was counted by SAMtools (6), and the assembly was computed with Flye v2.9 (7). The resulted contigs were polished using Racon v. 1.3.2 (8) (four iterations with parameters -m 8 -x -6 -g -8 -w 500) in combination with Medaka v. 1.4.3 (https://github.com/nanoporetech/medaka). The software default parameters were used except where otherwise noted. The assembly was circularized upon submission to the NCBI database. The quality of the genome assembly was assessed using QUAST v 5.1.0 (9). The genome sequence of *P. amylolyticus* W018 was assembled into a single circular chromosomal contig of 7,070,395 bp with an average GC content of 45.79%. The assembly was annotated using Prokaryotic Genome Annotation Pipeline (PGAP) v6.5 (10). The PGAP annotation identified 5,616 coding DNA sequences and 142 RNA sequences in the assembly (102 tRNAs, 36 complete rRNAs, and 4 noncoding RNAs). Notably, PGAP reports that the number of pseudogenes is about 30% of the gene features (2,432 pseudogenes out of 8,048 coding DNA sequences). These are probably caused by indels, which are typical for nanopore sequencing-only assemblies (11).

Genome similarity metrics (ANIb, FastANI, orthoANIb, and orthoANIu) were calculated using the gcType portal (12). The results of the genome analysis showed that the genome of strain W018 had high similarity with GCA_004001025.1 *P. amylolyticus*. Biosynthetic gene clusters for antimicrobial secondary metabolites were identified using antiSMASH 7.0 (13). The genome of strain W018 was predicted to encode antibiotics (paenilarvins A, B, and C; polymyxin; zwittermicin A), opine-type metallophores (bacillopaline), and lasso peptides (paeninodin).

## Data Availability

This project has been deposited at GenBank under accession no. PRJNA991090. The raw sequencing reads were deposited in the Sequence Read Archive under accession no. SRR25131389. The draft genome sequence accession number is CP130152.
